# Musculoskeletal Injuries and Conditions Among Homeless Patients

**DOI:** 10.5435/JAAOSGlobal-D-21-00241

**Published:** 2021-11-18

**Authors:** Nisha N. Kale, James Marsh, Neel K. Kale, Cadence Miskimin, Mary K. Mulcahey

**Affiliations:** From the Tulane University School of Medicine, (Ms. N. N. Kale, Mr. Marsh, and Mr. N. K. Kale), and the Department of Orthopaedic Surgery (Ms. Miskimin and Dr. Mulcahey), Tulane University School of Medicine, New Orleans, LA.

## Abstract

**Introduction:**

The purpose of this study was to analyze existing literature on musculoskeletal diseases that homeless populations face and provide recommendations on improving musculoskeletal outcomes for homeless individuals.

**Methods:**

A comprehensive search of the literature was performed in March 2020 using the PubMed/MEDLINE (1966 to March 2020), Embase (1975 to April 2020), and CINHAL (1982 to 2020) databases. The Preferred Reporting Items for Systematic Reviews and Meta-Analyses guidelines were used for accuracy of reporting, and the Newcastle-Ottawa Scale was used for quality assessment.

**Results:**

Twenty-nine articles met inclusion criteria. Seven studies observed an increased prevalence of musculoskeletal injuries among the homeless population, four observed increased susceptibility to bacterial soft-tissue infection, four observed increased fractures/traumatic injuries, three described increased chronic pain, and six focused on conditions specific to the foot and ankle region.

**Discussion:**

Homeless individuals often have inadequate access to care and rely on the emergency department for traumatic injuries. These findings have important implications for surgeons and public health officials and highlight the need for evidence-based interventions and increased follow-up. Targeted efforts and better tracking of follow-up and emergency department usage could improve health outcomes for homeless individuals and reduce the need costly late-stage interventions by providing early and more consistent care.

Musculoskeletal diseases and traumatic orthopaedic injuries are one of the leading causes of disability for adults in the United States, with homeless individuals at higher risk .^1^ A cohort study conducted among approximately 9,000 homeless men in Toronto from 1995 to 1997 found that men aged 25 to 44 years had a three times higher rate of mortality from being struck by a motor vehicle than the general population of Toronto. In those aged 45 to 64 years, the mortality rate was more than 4.5 times higher.^2^

Despite the high risk, very little literature exists on the prevalence and type of musculoskeletal injuries in homeless individuals. In 2014, Kay et al^3^ examined homeless patients presenting with orthopaedic trauma injuries to the emergency department (ED) and found that they were more likely to use the ED for orthopaedic follow-up and had poorer clinical follow-up rates compared with their nonhomeless counterparts. However, this study only accounted for orthopaedic trauma and not for other musculoskeletal injuries such as arthritis,^4^ podiatry-related issues including peripheral vascular disease and amputation,^5^ or advanced osteoarthritis of the hip and knee.^6^ In 1990, Gelberg et al reported that in a study comparing homeless and nonhomeless patients at a free clinic, homeless patients reported a higher degree of foot pain than nonhomeless patients.^4,7^

It is important to ascertain the prevalence of common orthopaedic injuries among homeless patients compared with nonhomeless patients and the reasons for these disparities.^7^ Musculoskeletal conditions may affect homeless individuals at a higher rate because of a higher prevalence of contributing factors such as chronic health problems, histories of physical or sexual abuse, and substance abuse disorders.^4^ Furthermore, Baggett et al^8^ reported in 2010 that among respondents to a Health Care for the Homeless User Survey, 73% experienced at least one unmet health need such as a lack of access to surgical care or dental care. Healthcare providers should have more information regarding the prevalence of musculoskeletal conditions in homeless individuals, which will help physicians provide better care to them. The purpose of this systematic review was to examine the existing literature and relevant data on musculoskeletal diseases and injuries in homeless population. We hypothesized that across these studies, homeless patients will have a greater burden of musculoskeletal injuries because of circumstantial factors such as inability to access care and environmental harms. In addition, we predicted that these injuries will be further complicated by systemic barriers to adequate care that this population face.

## Methods

### Overview

A systematic review was conducted in March 2020 according to the Preferred Reporting Items for Systematic Reviews and Meta-Analyses guidelines^9^ to identify specific articles dealing with musculoskeletal injuries in homeless populations. This review was registered with PROSPERO (Reg #:CRD42020176257). A protocol of the review has not been previously published. Quality assessment was conducted through the Newcastle-Ottawa Scale (NOS).

### Eligibility Criteria

All original research articles that reported on any and all musculoskeletal conditions that affect muscles, bones and joints, and associated tissues such as tendons and ligaments in homeless persons were included in this review. Conditions analyzed included but were not limited to tendinitis, carpal tunnel syndrome, osteoarthritis, rheumatoid arthritis, fibromyalgia, and fractures. Articles that did not present clear data on orthopaedic musculoskeletal injuries in homeless persons were excluded. This included the following categories: reviews, case reports, commentaries, and dissertations.

### Information Sources and Search Strategy

**Figure 1:**
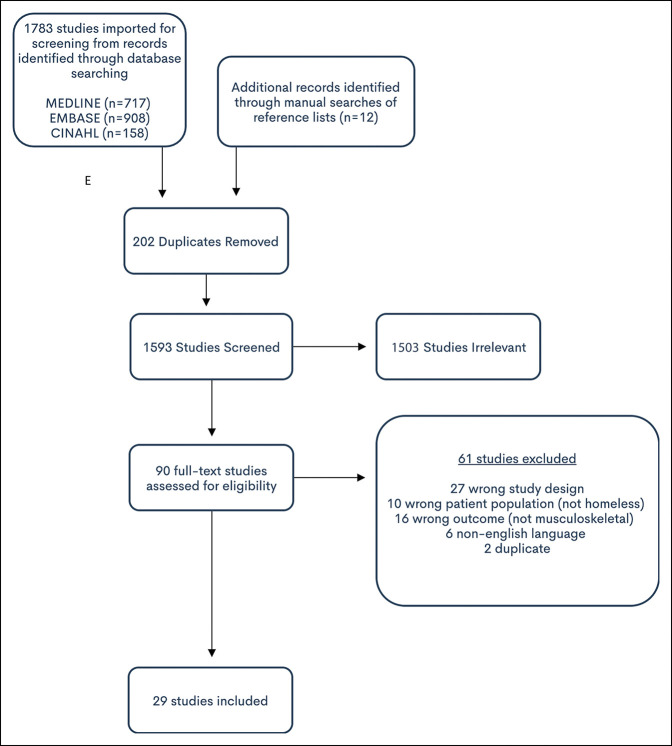
Flowchart showing criteria used for Preferred Reporting Items for Systematic Reviews and Meta-Analyses search. CINAHL = cumulative index to nursing and allied health literature, MEDLINE = medical literature analysis and retrieval system online, Embase = excerpta medica database

Articles were identified through a search of MEDLINE (PubMed) (1966 to 2020), Embase (1947 to 2020), and CINHAL (1982 to 2020) with English language exclusions. The search strategy was created by a member of the research team in consultation with a health sciences librarian and included terms related to homelessness and crossmatched with common musculoskeletal and orthopaedic health terms (Figure 1). This search was complemented by manual searches of reference lists from research articles that were relevant to the topic. The search concluded in July 2020.

### Study Selection

Titles and abstracts of potentially suitable articles were screened using Covidence systematic review software^10^ by three independent reviewers, one of whom is a board-certified orthopaedic surgeon. In case of ambiguity, the full-text article was retrieved to determine its eligibility, and the final decision was deferred to the senior author. Articles that met inclusion criteria were included in full-text analysis. Disagreements or discrepancies in the screening process were resolved by consensus voting.

### Data Extraction and Analysis

Data were extracted by two independent reviewers based on the following variables: study characteristics (including primary author, year of publication, country of study, and source of funding); participant demographics (age, sex, race if available, and socioeconomic data if available); study design and setting; musculoskeletal conditions identified; anatomical location(s) if available; and assessment of musculoskeletal condition; and study findings. Quality assessment was performed by two independent reviewers using the NOS.^11^ An adapted version of the NOS was used to evaluate cross-sectional studies^12^ and case series.^13^ The scale assesses selection, comparability, and outcome of studies. Quality assessment breakdown is summarized in Tables 2–5.

## Results

The initial search yielded 1,795 articles. After screening titles and abstracts, 90 studies remained. The full text of all articles was reviewed and screened to determine eligibility for the study. Of these, 59 studies were excluded because they did not include data that presented clear information on musculoskeletal conditions and outcomes, have the correct study design, or include homeless populations (Figure 1). Twenty-nine studies met all inclusion criteria and were included in the systematic review (Table 1).

**Table 1:** Characteristics of the 29 Included Studies

First Author (year)	Sample Size (% male); % Homelessness	Race % Homeless (if available)	Mean Age (range)	Study Setting	Assessment of Musculoskeletal Condition	Study Findings	QI Score
Barshes (2016)^14^	N = 184 (4.9% homeless)	NR	65	Michael E. DeBakey Veterans Affairs Medical Center, Houston, TX	Chart review	For the treatment of foot osteomyelitis, homelessness associated with time to treatment failure (moderate additional risk of +3 [of 4], *P* < 0.001) and amputation. (*P* = 0.002).	4
Bennett (2017)^15^	N = 33 (94%) (100%)	NR	53 (38-74)	VA Palo Alto Health Care System	Chart review	Total joint arthroplasty: total knee arthroplasty (n = 18), total hip replacement (n = 18), and unicondylar knee replacement (n = 1).	5
Chen (2014)^16^	N = 299, (92.0%) (100%)	61% Black	62% ranged from 36 to 55	Two homeless shelters in San Francisco, California	Questionnaire	In the sample of 299 participants, self-reported foot problems included foot pain (56%), fungal nail (30%), previous foot injuries (27%), calluses (26%), athlete's foot (24%), and corns (19%). Other conditions included ingrown nails (15%), bunion (14%), hammertoe (7%), gout (6%), immersion foot (5%), ulcers (4%), warts (4%), peripheral artery disease (3%), type 2 diabetes mellitus (5%), type 1 diabetes mellitus (4%), and frostbite (2%).	8
Chong (2014)^17^	N = 95 (75%) (100%)	NR	Male, 49 (11.3)	Long Beach California WISH/MASH, gender based programs which provide community-based services to the homeless, sponsored by the American University of Health Sciences	Questionnaire	37% of individuals reported having foot problems (pain, sores, or bleeding), 31% reported arthritis, and 25% reported taking over-the-counter NSAID medications and acetaminophen for joint and muscle pain.	4
Ferenchick (1992)^18^	N = 475, (66.95%) (38.10%)	61% White, 28% Black	34.3	A community health clinic located in Lansing, Michigan	Interview, clinical examination	Of the 181 homeless individuals in this study, 47 (26%) presented with a musculoskeletal injury. Of these, 13% were soft-tissue injuries, and 11% were fractures. Homeless individuals were significantly seen for injuries/fractures (*P* < 0.05) than individuals with stable and unstable housing.	7
Field (2019)^19^	N = 1135, (77.0%) (100%)	NR	43 (SD = 22.8)	“Pathway” homelessness teams in seven UK hospitals	Chart review	18.6% sustained external injuries including road traffic accidents, assault, trauma, animal bite, and deliberate self-harm; 8.2% sustained abscess, cellulitis, and dermatitis; and 5.3% sustained diseases of the musculoskeletal system and connective tissue (osteomyelitis, septic arthritis, limb swelling, dislocation, and back pain).	5
Frencher (2010)^20^	N = 1,528,695 (42.9%) (21.33%)	10.0% White, non-Hispanic, 36.9% Black, non-Hispanic, 7.7% Hispanic, 45.4% other/unknown	N/A	Multiple hospitals in NYC	Chart review	Compared with low SES housed patients, unintentional injury among the homeless individuals was 13% higher in children, 6% higher in adults, and 63% higher in elderly adults. Compared with low SES housed, all groups aged ≥10 years experienced significantly increased odds of assault-related hospitalization. Hospitalizations for assault were more than threefold higher in homeless elderly cohort compared with low SES housed elderly population.	8
Goldstein (2008)^21^	N = 2978 (97%) (100%)	46.9% White, 51.6% AA, 1.5% other	49	Veterans Integrated Services Network 4 that includes all of Pennsylvania and Delaware and parts of West Virginia, New York, and Ohio.	Interview and chart review	Dental and orthopaedic problems were the most common reports; 1552 orthopaedic disorders in veteran patients (52.1%).	4
Howell (2016)^6^	N = 169 (male:female ratio of 1.4:1) (47% homeless)	NR	43 (1-91)	University College London Hospitals NHS Foundation Trust, UK	Chart review	9% of patients who had a suspected bone, native joint, or soft-tissue infection were classified as homeless or unstably housed. Within the total sample, the most common diagnoses were cellulitis, abscesses, septic arthritis, and osteomyelitis.	4
Jetelina (2017)^22^	N = 132 (78%) (22.2%)	White NH (39%); Black NH (35%); Hispanic (17%); other (9%)	36.9 (11.5)	Baylor University Medical Center, Dallas; Methodist Dallas Medical Center; University Medical Center Brackenridge, Austin	Clinical examination/interview	39% of homeless adults were treated for violent injury. Homeless adults had 1.67 higher odds of intentional violent injury, and 1.95 higher odds of stabbing injury. For intentional injury, 33% were struck by/against or crushed, 25% gunshot wound injury, and 42% stabbed.	5
Jones (1990)^23^	N = 511 (82.6%) (100%)	75% Black, 16% White, 6% Hispanic, 3% AI	Female: 37.8 (23-77); male: 43.7 (21-75)	5 homeless shelters in Chicago	Clinical Examination/interview	Conditions seen in 511 patients with 900 visits: tylomata (callus), 85; unguis incarnatus (ingrown toenails), 78; ulcers, 32; symptomatic hallux valgus, 25; idiopathic painful edema, 25; sprained ankles, 24; peripheral neuropathy, 22; arch pain, 21; trauma (self-generated), 13; fractures (digital, metatarsal, or fibular), 13; heel pain, 11; bursitis/ganglion/capsulitis, 10; chilblains, 10; ankle pain, 10; fissured heels, 9; entrapment neuropathy (footdrop), 8; frostbite, 8; foreign body (glass, nail, or splinter), 6; tendinitis, 5; symptomatic digiti quinti varus, 5; symptomatic contracted toes (no keratoses), 5; residual pain from previous surgery or trauma, 5; cellulitis, 3; gout, 2; and burn, 1.	8
Kay (2014)^3^	N = 126 (89.7%) (50%)	NR	46.6	Vanderbilt University Medical Center ED	Chart review	Homeless orthopaedic patients sustained more upper extremity trauma (41.3%) and spine trauma (12.7%) than nonhomeless patients, and more infection (9.5%), nonunion (4.8%), and hardware failure (4.8%), although these differences were not statistically significant compared with nonhomeless patients. Of the homeless patients, 91% received an ED consult compared with 9% of the nonhomeless patients (*P* = 0.15). Homeless patients had more ED visits and fewer orthopaedic clinic follow-up visits than nonhomeless patients (*P* = 0.001).	6
Kleinman (1996)^24^	N = 363 (70%) (100%)	61% Black, 22% White 8% Latino	37.6	Field survey of homeless adults in Los Angeles County, California	Interview, clinical examination	Of the 363 respondents who were interviewed and examined, 24% (358) self-reported foot abnormalities and foot conditions. 18% (N = 270) were found to have foot abnormalities after physical examination, and 97 patients (25%) with foot abnormalities were referred to orthopaedic surgeons or podiatrists	8
Kornblith (2013)^25^	N = 201 (22.9% homeless)	NR	53.8 (18.7)	San Francisco General Hospital	Chart review	46 “found down” patients (22.9%) were homeless. Of the 201 found down patients, 8 (19.5%) were triaged to the ED trauma bay and 38 (23.8%) to primary evaluation by the medical service.	6
Kowal-Vern (2007)^26^	N = 1615 (4.5% homeless)	African American, 59% versus 68%; Hispanic, 25% versus 10%; Caucasian, 12% versus 20%; and other, 4% versus 2% (*P* = 0.03)	44	Sumner L. Koch Burn Center, Department of Trauma, John H. Stroger, Jr., Hospital of Cook County, Chicago, Illinois	Chart review	Frequency of burn injury among the homeless: Flames (44%), frostbite (29%), scald (14%), and contact injury (9%). Significantly, more homeless individuals were admitted for frostbite (*P* < 0.001).	4
Laere (2009)^10^	N = 629, (83.0%) (100%)	53% Caucasian	45 years (SD 10 years)	A shelter-based convalescence care facility (Gutenberg) in Amsterdam	Interview, clinical examination	165 of 629 (26%) homeless adults were admitted with musculoskeletal (locomotion) conditions. Of these conditions, 19% were identified as injuries and 6% as fractures.	7
Landefeld (2017)^4^	N = 348 (77.3%) (100%)	79.6% African American	Median age = 58 (50-80)	Homeless adults from overnight shelters, homeless encampments, meal programs, and a recycling center in Oakland, California.	Questionnaire	Homeless adults reported arthritis, 154 (44.3%); history of abuse, 272 (75.3%); moderate pain over the past week (17.2%), severe pain over the past week (39.4%), chronic moderate to severe pain (46.8%), and chronic pain (79.9%).	5
Lee (2007)^27^	N = 76 (28.9%) (100%)	Chuukese (84%), Marshallese (13%), Pohnpeian (1%), Kosraean (1%)	23	Hawaii H.O.M.E. Project student-run free medical clinic, Honolulu, HI	Questionnaire	For preexisting conditions, musculoskeletal was most commonly reported (back trouble [14], arthritis [9], and fractures [5]). Musculoskeletal reports were a common assessment (8.6%, include patello-femoral syndrome, knee pain, neck strain, chest wall pain, plantar fasciitis, shoulder strain, costochondritis, neck strain, and sciatica)	4
Mackelprang (2014)^28^	N = 268 (80%) (100%)	N/A	Male: 43.3 years, SD = 13.9, female: 38.3 years, SD = 2.2	Data were pulled from the Consumer Product Safety Commission operated NEISS, a database of consumer product-related injuries from a stratified national probability sample of 100 US ED.	Chart review	The most common injury diagnosis among homeless individuals and control subjects was sprain/strain (55 [20.5%] and 647 [24.1%], respectively); 50.9% were associated with the trunk, usually the back, because of carrying heavy objects, falling, or sleeping on hard surfaces. 5.59% (15) had fractures.	4
Mosites (2018)^29^	N = 90 (64.4%) (43.33%)	74% Alaskan Native	52	Hospitals in Anchorage, Alaska	Chart review, antibiotic intervention	For patients with an emm26.3 strain of group A *Streptococcus* sp. (43), 81% were homeless (35), 63% (27) presented with cellulitis, 49% (21) with sepsis, 4% (2) with streptococcal toxic shock syndrome, 23% (10) with necrotizing fasciitis, 12% (5) with pneumonia, and 5% (2) with septic arthritis.	5
Murata (1992)^30^	N = 303 (100% homeless)	24% Black, 2% Asian	8.46	UCLA School of Nursing Health Center at Union Rescue Mission	Examination	78.6% of homeless children had lacerations and open wounds compared with 64.1% of the children control group, and 21.4% had sprain and strains compared with 35.9% of the children control group.	6
Oosman (2019)^31^	N = 47 (70.2%) (100%)	NR	47 (21-72)	Clients of the Lighthouse Supported Living facility in Saskatoon, Saskatchewan, Canada	Questionnaire	Most homeless clients in the sample, 85.1% (40), presented with an orthopaedic issue causing chronic pain or decreased mobility in them.	4
Pearson (2007)^32^	N = 600 (50% homeless)	46% Hispanic, 15% Black, 2% Asian	36 (IQR 25-46)	Denver Health Medical Center	Chart review	14% of homeless persons presented with laceration, 9% with contusion, hematoma or abrasion, and 8% with fracture, dislocation, or subluxation. The most common diagnoses included laceration (13%) and fracture-dislocation or subluxation (5%).	6
Robbins (1996)^33^	N = 81	89.8% Black, 10.2% White	38.6 (11-74)	One-day clinic and survey operated by Department of VA, North East OH Coalition for Homeless, Catholic Diocese of Cleveland	Interview, Clinical examination	More individuals sustained foot abnormalities, including ulcers/corns and severe athlete's foot (358, 24%)	5
Takano (1999)^34^	N = 1938 (100% [100%])	100% Japanese	34.5	Ichiji-hogo-soudan-syo, an institution operated by the Human and Health Affairs Union of the city-wards of the Tokyo Metropolis in Japan	Examination	29.9% of the sample presented with fractures, dislocations, sprains, and strains, 49.6% of whom were former construction workers. 89.3% of the sample presented with dorsopathies. Morbidity for homeless individuals was three times higher than the general population for dorsopathies and fractures, dislocations, sprains, and strains.	3
Thapa (2009)^35^	N = 48 (95.8%) (100%)	100% Nepalese	68.8% (N = 33) ranged from 11-15 years of age	Laboratory and physical examinations performed at the BP Koirala Institute of Health Sciences, in Dharan Municipality, Nepal	Clinical examination	27 of 48 children reported health problems involving extremities, including joint pain (15, 31.2%) and cramps (12, 25%). Overall, 56.25% of the sample reported health problems with extremities.	6
Vindigni (2011)^36^	N = 290 (preintervention), N = 192 (postintervention); N = 66 analyzed for pre-post treatment analysis (100% homeless)	100% Filipino	NR	Hands on Philippines education clinic that serves the homeless community in Bagong Barrio, Caloocan, Philippines	Questionnaire	Pretreatment patients reported pain in upper back (36.7%), lower back (18.7%), shoulders (16.3%), hips/thighs (3.0%), wrists (1.2%), and elbows (0.6%). 50% experienced an average of 5 musculoskeletal conditions.	7
Young (2004)^37^	N = 6,156 (84% homeless)	n = 2,542 (41%) White, n = 1,907 (31%) Black, n = 1,150 (19%) Hispanic	42 years (range 1-89)	The San Francisco General Hospital Integrated Soft Tissue Infection (ISIS) Clinic	Chart review	695 cultures (83%) contained Staphylococcus *aureus*, and 525 cultures (63%) contained MRSA. Homeless patients had an OR of 1.4 for *S aureus* compared with patients with stable living situations (*P* = 0.04). Incision and drainage of an abscess or débridement of the wound was used to treat most infections (64%).	5
Zuccaro (2018)^38^	N = 97 (79%) (100%)	NR	46.7	The Ottawa Hospital Emergency Department	Chart review	Of the 83 surgical referrals for traumatic injuries, 66 (80%) were for fractures: 46 patients (70%) were sent to orthopaedic surgery. In almost two-thirds (42 [64%]) of surgical referrals for fractures, patients did not complete treatment and were lost to follow-up; 30 (65%) of those lost to follow-up were referred to orthopaedic surgery.	4

ED = emergency departments, NR = not reported, NHS = National Health Service, NH = Non-Hispanic, AA = African-American, NEISS = National Electronic Injury Surveillance System, IQR = Interquartile Range

### Setting

Of the 29 studies screened, 21 studies (72%) were conducted in the United States, two (7%) in the United Kingdom,^6,19^ two (7%) in Canada,^31,38^ and one each in Japan,^34^ Amsterdam,^10^ Nepal,^35^ and the Philippines.^36^ Fourteen of the 29 studies (48%) collected data on homeless patients from hospitals and ED settings.^3,6,14,15,19,20,22,25,26,28,29,32,37,38^ Shelters,^4,10,16,30,31,34^ community health clinics,^18,23,27,36^ and field surveys^17,24,33^ were also used. Of the six prospective studies included in the analysis, two recruited homeless individuals from shelters,^10,23^ two recruited those directly from the community through field surveys,^24,36^ one recruited those from a community health clinic,^18^ and one recruited self-identified homeless individuals from a hospital ED.^22^ Twenty-four studies (83%) recruited and collected data from participants at one location, whereas five studies (17%)^19,22,28^ were conducted across multiple hospitals and two studies^16,23^ across multiple shelters.

### Sample

The median number of participants in our sample was 279, with a range of 47^31^ to 1,528,695 participants.^20^ Twenty-five studies (86%) reported the age of participants, whereas four studies did not provide the mean age of participants.^16,20,35,36^ The average reported mean age of participants was 43 years (range, 8.46 to 65 years). Two studies^23,28^ reported the stratified mean age of male and female participants. One study had only juvenile participants,^30^ and three studies stratified their sample by adult and juvenile participants.^20,28,30,35^ Most studies had mainly male participants (range, 42.9%^20^ to 100%^34^), although eight studies did not report gender of their participants. Seventeen studies (59%) comprised only homeless individuals (range, sample size N=33 to N=2,978). Fourteen studies (48%) formally defined homelessness in their articles, although definitions were not well-defined and differed widely between studies, whereas 15 studies (52%) did not. Duration of homelessness was provided only in three studies,^10,24,34^ with no information on participants’ moves or housing transitions. Twenty studies (69%) provided information regarding participants' ethnic or racial background, most of which were from minority backgrounds. In nine studies (31%), the sampled population was mostly Black or African American, and in three studies (10%), the sampled population was mostly White.^16,22,37^ No studies assessed differences in musculoskeletal conditions or outcomes by race. Twelve studies (41%) reported information regarding health insurance coverage rates (eg, Medicare/Medicaid coverage rates^20^) among homeless participants.

### Funding

Six studies (21%) were supported by a government agency, and two studies (7%) were supported by a nongovernmental source of funding. One study was supported by a governmental and nongovernmental funding.^37^ Twenty studies (69%) did not indicate any source of funding.

### Study Design and Musculoskeletal Assessment

Most studies (N = 13, 45%) were descriptive in nature and were retrospective chart reviews of health clinics and EDs in hospitals that served homeless individuals. Ten studies (34%) involved a clinical examination and subsequent diagnosis of presenting musculoskeletal condition. Six studies (20%) involved questionnaires that recorded subjective descriptions of individuals health and any reports regarding their own health status, such as the prevalence and location of pain,^4,36^ self-reported history of preexisting medical conditions,^27^ and current medical reports.^16,17,31^ Only one study^10^ was a longitudinal, 7-year descriptive study analyzing the diagnoses and use of shelter-based convalescence care facilities for homeless individuals.^27^ Three prospective studies involved specific interventions for homeless individuals, including antibiotic intervention for soft-tissue staphylococcal infection,^37^ physical therapy and pain management for self-reported pain,^36^ and referral to orthopaedic surgeons or podiatrists for self-reported abnormalities and foot conditions.^24^ One cross-sectional study included education on proper foot care and distribution of footwear.^16^

### Methodological Quality

The overall methodological quality of the studies was moderate with a median score of 5 (range, 3 to 8), which was determined using the NOS (Tables 2–5). Of the 29 studies reviewed, two (7%) were case-control studies. Both studies were of high quality, receiving scores of at least seven (Table 2). Eleven cross-sectional studies were reviewed (Table 3). Each of these studies provided an explanation for assessment of the outcome, but no descriptions were provided for control of confounding factors. There were 16 cohort studies (Table 5), which were representative of the average homeless individuals in the community. Every study provided an assessment of the outcome. One case series was reviewed (Table 4) and was of low quality, with a score of 3.^15^

**Table 2:** Quality Assessment Using the Newcastle-Ottawa Scale: Case-Control Studies

Case-Control Studies	Mackelprang (2014)	Barshes (2016)
Is the case definition adequate? a) Yes, with independent validation Ø (Requires some independent validation (eg, >1 person/record/time/process to extract information, or reference to primary record source such as radiographs or medical/hospital records)* b) Yes, eg, record linkage or based on self-reports (record linkage [eg, ICD codes in database] or self-report with no reference to primary record) c) No description	1	1
2) Representativeness of the cases: a) All eligible cases with outcome of interest over a defined period, all cases in a defined catchment area, all cases in a defined hospital or clinic, group of hospitals, health maintenance organization, or an appropriate sample of those cases (eg, random sample).* b) Not satisfying requirements in part (a) or not stated	1	1
3) Selection of controls: This item assesses whether the control series used in the study is derived from the same population as the cases and essentially would have been cases had the outcome been present: a) Community controls (ie, same community as cases and would be cases if had outcome)* b) Hospital controls, within same community as cases (ie, not another city) but derived from a hospitalized population c) No description	1	1
4) Definition of controls: a) If cases are the first occurrences of outcome, then it must explicitly state that controls have no history of this outcome. If cases have new (not necessarily first) occurrence of outcome, then controls with previous occurrences of outcome of interest should not be excluded.* b) No mention of history of outcome	0	0
Selection sum (max. 4 stars)	3	3
Comparability: A maximum of 2 stars can be allotted in this category. Either cases and controls must be matched in the design or confounders must be adjusted for in the analysis. Statements of no differences between groups or that differences were not statistically significant are not sufficient for establishing comparability. Note: If the odds ratio for the exposure of interest is adjusted for the confounders listed, then the groups will be considered to be comparable on each variable used in the adjustment. There may be multiple ratings for this item for different categories of exposure (eg, ever versus never, current versus previous or never; study controls for age or study controls for comorbidities).	2	1
Comparability (max. 2 stars)	2	1
1) Ascertainment of exposure: a) Secure record (eg surgical records)* b) Structured interview where blind to case/control status* c) Interview not blinded to case/control status d) Written self-report or medical record only e) No description	1	1
2) Same method of ascertainment for cases and controls: a) Yes* b) No	1	1
3) Nonresponse rate: a) Same rate for both groups* b) Nonrespondents described c) Rate different and no designation	1	1
Exposure sum (max. 3 stars)	3	3
Total QI score (max. 9 stars)	8	7

Asterisk indicates how many points are given if a study falls within that category (* = 1 point, ** = 2 points, no asterisk means no points).

**Table 3:** Quality Assessment Using the Newcastle-Ottawa Scale: Cross-Sectional Studies

Cross-sectional Studies	Lee (2007)	Jones (1990)	Thapa (2009)	Goldstein, 2008	Chong (2014)	Murata (1992)	Oosman (2019)	Landefeld (2017)	Robbins, 1996	Chen (2014)	Field (2019)
1) Representativeness of the sample: a) Truly representative of the average in the target population* (all subjects or random sampling) b) Somewhat representative of the average in the target population* (nonrandom sampling) c) Selected group of users d) No description of the sampling strategy	0	1	0	1	1	1	1	1	1	1	1
2) Sample size: a) Justified and satisfactory* b) Not justified	0	1	0	1	1	1	1	1	1	1	1
3) Nonrespondents: a) Comparability between respondents and nonrespondents characteristics is established, and the response rate is satisfactory.* b) The response rate is unsatisfactory, or the comparability between respondents and nonrespondents is unsatisfactory. c) No description of the response rate or the characteristics of the responders and the nonresponders	0	1	0	0	0	1	0	0	1	1	1
4) Ascertainment of the exposure (risk factor): a) Validated measurement tool** b) Nonvalidated measurement tool, but the tool is available or described* c) No description of the measurement tool	1	0	1	1	1	1	2	2	2	1	1
Selection sum (max. 5 stars)	1	3	1	3	3	4	4	4	5	4	4
Comparability: (max. 2 stars) 1) The subjects in different outcome groups are comparable, based on the study design or analysis. Confounding factors are controlled. a) The study controls for the most important factor (select one).* b) The study controls for any additional factor.*	0	0	0	0	0	0	0	0	0	0	0
Comparability sum (max. 2 stars)	0	0	0	0	0	0	0	0	0	0	0
1) Assessment of the outcome (max 3 stars): a) Independent blind assessment** b) Record linkage** c) Self-report* d) No description	3	1	3	3	1	2	3	1	1	1	2
2) Statistical test: a) The statistical test used to analyze the data is clearly described and appropriate, and the measurement of the association is presented, including confidence intervals and the probability level (*P* value).* b) The statistical test is not appropriate, not described, or incomplete.	0	1	0	0	0	0	0	1	0	0	1
Outcome (max. 3 stars)	3	2	3	3	1	2	3	2	1	1	3
Total QI score (max. 10)	4	5	4	6	4	6	7	6	6	5	7

Asterisk indicates how many points are given if a study falls within that category (* = 1 point, ** = 2 points, no asterisk means no points).

**Table 4:** Quality Assessment Using the Newcastle-Ottawa Scale: Case Series

Case Series (4)	Bennett, 2017
1) Is the case definition adequate? a) Yes, with independent validation* b) Yes, eg, record linkage on self-reports c) No description	1
2) Representativeness of the cases: a) Consecutive or obviously representative series of cases* b) Potential for selection biases or nonstated	1
3) Selection of controls: a) Community controls* b) Hospital controls c) No description	0
4) Definition of controls: a) No history of disease (end point)* b) No description of source	0
Selection sum (max. 4 stars)	2
1) Comparability of cases and controls on the basis of design or analysis: a) Study controls for age and education* b) Study controls for any additional factors*	0
Confounder sum (max. 2 stars)	0
1) Ascertainment of exposure: a) Secure records* b) Structured interview where blind to case/control status* c) Interview not blinded to case/control status d) Written self-reports or medical record only e) No description	1
1) Same method of ascertainment for cases and controls: a) Yes^a^ b) No	0
2) Nonresponse rate: a) Same rate for both groups* b) Nonrespondents described c) Rate different and no designation*	0
Exposure sum (max. 3 stars)	1
Total QI score (max. 9)	3

Asterisk Indicates how many points are given if a study falls within that category (* = 1 point, ** = 2 points, no asterisk means no points).

**Table 5:** Quality Assessment Using the Newcastle-Ottawa Scale: Cohort Studies

Cohort Studies (3)	Ferenchick, G (1992)	Mosites, E (2018)	Young (2004)	Takano (1999)	Kornblith (2013)	Kleinman, L (1996)	Beijer, U (2009)	Pearson, D.A (2007)	Jetelina (2017)	Zucaro (2018)	Frencher (2010)	Kay (2014)	Kowal-Vern (2007)	Howell (2016)	Laere (2009)	Vindigni (2011)
1) Representativeness of the exposed cohort: a) Truly representative of the average homeless individuals (describe) in the community* b) Somewhat representative of the average homeless individuals in the community* c) Selected group of users, eg, nurses and volunteers d) No description of the derivation of the cohort	1	1	1	1	1	1	1	1	1	1	1	1	1	1	1	1
2) Selection of the nonexposed cohort: a) Drawn from the same community as the exposed cohort* b) Drawn from a different source c) No description of the derivation of the nonexposed cohort	0	1	1	1	1	0	1	1	1	0	1	1	1	1	1	1
3) Ascertainment of exposure: a) Secure record (eg, surgical records)* b) Structured interview* c) Written self-report	1	1	1	1	1	1	1	1	1	1	1	1	1	1	1	1
4) Demonstration that outcome of interest was not present at the start of Study In the case of mortality studies, outcome of interest is still the presence of a disease/incident, rather than death, which means a statement of no history of disease or incident earns a star.	0	0	0	0	0	0	0	0	0	0	0	0	0	0	0	0
Selection sum (max. 5 stars)	2	3	3	3	3	2	3	3	3	2	3	3	3	3	3	3
Comparability 1) Comparability of cohorts on the basis of the design or analysis. A maximum of 2 stars can be allotted in this category. Either exposed and nonexposed individuals must be matched in the design or confounders must be adjusted for in the analysis. Statements of no differences between groups or that differences were not statistically significant are not sufficient for establishing comparability. Note: If the relative risk for the exposure of interest is adjusted for the confounders listed, then the groups will be considered to be comparable on each variable used in the adjustment. There may be multiple ratings for this item for different categories of exposure (eg, ever versus never, current versus previous or never; study control subjects for age or study control subjects for comorbidities).	0	0	0	2	0	0	2	0	2	0	2	2	0	0	2	0
Comparability sum (max. 2 stars)	0	0	0	2	0	0	2	0	2	0	2	2	0	0	2	0
1) Assessment of outcome: For some outcomes (eg, fractured hip), reference to the medical record is sufficient to satisfy the requirement for confirmation of the fracture. This would not be adequate for vertebral fracture outcomes where reference to radiographs would be required. a) Independent or blind assessment stated in the article or confirmation of the outcome by reference to secure records (radiographs, medical records, etc.)* b) Record linkage (eg, identified through ICD codes on database records) c) Self-report (ie, no reference to original medical records or radiographs to confirm the outcome) d) No description	1	1	1	1	1	1	1	1	1	1	1	1	1	1	1	1
2) Was follow-up long enough for outcomes to occur? An acceptable length of time should be decided before quality assessment begins (eg, 5 yr for exposure to breast implants).	0	1	0	0	0	1	1	1	1	0	1	1	0	0	1	0
3) Adequacy of follow-up of cohorts: a) Complete follow-up—all subjects accounted for* b) Subjects lost to follow-up unlikely to introduce bias—small number lost: ____ % (select inadequate %) follow-up, or description provided of those lost)* c) Follow-up rate: ____% (select an adequate %) and no description of those lost d) No statement	1	0	0	0	0	1	0	0	0	1	1	1	0	0	1	1
Outcome sum (max. 5 stars)	2	2	1	1	1	3	2	2	2	2	3	3	1	1	3	2
Total QI score (max. 12)	4	5	4	6	4	5	7	5	7	4	8	8	4	4	8	5

Asterisk Indicates how many points are given if a study falls within that category (* = 1 point, ** = 2 points, no asterisk means no points).

### Musculoskeletal Conditions

Seven manuscripts (24%) observed an increased prevalence of musculoskeletal injuries among the homeless, although the anatomic location and specific type of injury were not reported. Laere et al^28^ and Ferenchick et al^17^ both noted that 26% of reported injuries among the homeless were musculoskeletal in nature, including fractures, and Thapa et al^37^ noted that 31.2% homeless children sampled had extremity problems. Ferenchick et al^17^ specifically noted that homeless individuals were significantly seen for musculoskeletal injuries/fractures (*P* < 0.05) than individuals with stable and unstable housing. Two studies,^21,31^ which sampled elderly homeless individuals, noted that most reports involved orthopaedic and musculoskeletal issues. Two other studies found that musculoskeletal conditions involving back pain (18.4%)^27^ and back strains (50.9%)^28^ associated with the trunk because of carrying heavy objects, falling, or sleeping on hard surfaces were common reports among homeless individuals.

### Musculoskeletal Pathology and Infection

Four studies (14%) discussed pathology related to musculoskeletal conditions. Field et al^35^ and Howell et al^10^ found that within their samples, septic arthritis and osteomyelitis were common diagnoses, and 9% of homeless individuals had a suspected bone or joint infection.^6,19^ In 2018, Mosites et al^29^ examined an outbreak of group A streptococcal cases in Alaska and found that 81% of infections occurred in homeless individuals, 23% of whom presented with necrotizing fasciitis. Young et al^37^ examined the risk of methicillin-resistant *Staphylococcus aureus* soft-tissue infections and found that homeless patients had 1.4 higher odds for *S aureus* soft-tissue infection compared with patients with stable living situations (*P* = 0.04).

### Trauma

Four studies (14%) described the prevalence of fractures and traumatic injuries among homeless individuals. In 2018, Zuccaro et al^38^ found that 80% of surgical referrals for homeless patients with traumatic injuries were fractures and 70% of homeless patients were sent to orthopaedic surgeons. Takano et al^34^ found that morbidity for homeless individuals was three times higher than the general population for disorders of the spine, fractures, dislocations, sprains, and strains. Fractures caused by external injuries, such as road traffic accidents or assault (18.6%), were common injuries among homeless patients visiting UK hospital EDs.^19^ One study investigating patients who were “found down” or unresponsive with no clear mechanism of injury found that 22.9% of their sample were homeless individuals.^25^ Nearly half (42.8%) of the patients found down were injured, including intracranial, long-bone, facial, spinal, and pelvic fractures.

Homeless patients are at greater risk of traumatic injury due to violence. In 2017, Jetelina et al^22^ found that homeless adults had 1.67 higher odds of intentional violent injury and 1.95 higher odds of stabbing injury. Frencher et al^20^ found that hospitalizations for assault were more than three times higher in the homeless compared with those in the housed control group. Homeless orthopaedic patients particularly sustained more upper extremity trauma (41.3%) and spine trauma (12.7%) than nonhomeless patients and experienced more surgical complications including infection (9.5%), nonunion (4.8%), and hardware failure (4.8%).^3^

Homeless individuals are at greater risk of musculoskeletal injury because of the nature of being exposed to the elements and environmental injuries. In 2007, Kowal-Vern et al^26^ found that 29% of homeless individuals presented with frostbite and 44% with burn injures, which was significantly higher than the general population (*P* < 0.001). Another study noted that among homeless children specifically, 78.6% had lacerations and open wounds compared with the control group, likely because of being exposed to hazardous objects in their environment.^30^ Pearson et al^26,32^ found that the most common diagnoses among homeless adults presenting to the ED were laceration (13%) and fracture-dislocation or subluxation (5%).

### Pain Related to Musculoskeletal Conditions

Three studies (10%) detailed chronic pain related to musculoskeletal conditions. In 2014, Chong et al^17^ found that 25% of homeless individuals reported taking over-the-counter nonsteroidal anti-inflammatory medications for joint and muscle pain. Landefeld et al^4^ interviewed older homeless (range, 50 to 80 years) adults in Oakland, CA, and found that 44.3% reported arthritis and 79.9% reported having issues of chronic pain. Another study performed at a clinic serving a homeless community in the Philippines found that 50% of respondents experienced an average of five musculoskeletal conditions, with 36.7% reporting pain in the upper back.^36^

### Lower Extremity

Only one study examined how many homeless patients underwent a specific orthopaedic procedure. Bennett et al^15^ discussed the management of homeless patients who are total joint arthroplasty candidates and detailed how 37 successful primary joint replacement surgeries were performed on 33 homeless patients with advanced osteoarthritis. Foot pathology and injuries were also very common among homeless patients. Six studies (21%) evaluated injuries and conditions specific to the foot and ankle. Five studies (17%) that surveyed homeless individuals in the field (including at community outreach events and homeless shelters) found that foot problems (eg, abnormalities, pain, sores, or bleeding) were among the most common self-reported injuries,^16,17,23,24,33^ including specific conditions such as foot pain (56%) and previous foot injuries (27%),^16^ severe athlete's foot (24%),^33^ and arch pain (4.3%), sprained ankles (4.9%), ulcers (51.3%), and trauma (4.1%).^23^ In 2016, Barshes et al^14^ examined the management of foot osteomyelitis and found that homelessness was significantly associated with time to treatment failure (9.5 HR, *P* < 0.001) and major amputation (11.3 HR, *P* < 0.001).

## Discussion

Across studies that were reviewed, between 14% and 100% of homeless individuals reported orthopaedic concerns.^14,32^ Despite the numerous studies identified as part of this systematic review, much of the existing literature only offers a general overview of orthopaedic injuries without reporting specific anatomical locations of the injuries described. Precise anatomical locations of musculoskeletal issues were not provided in many of the studies, whereas others described problems in general regions of the body such as the back or extremities and were unable to adequately report the exact cause of the problem. Three studies identified specific locations including upper back, lower back, wrists, or hips in patients.^15,16,36^ Six studies^3,14,18,22,26,37^ found that homeless individuals were markedly seen for injuries or fractures compared with those with stable and unstable housing. Six studies (21%) found that homeless individuals were more susceptible to musculoskeletal injuries and fractures,^34^ musculoskeletal pathology and infections, trauma, pain related to musculoskeletal conditions,^20,28^ and assault-related injuries^1,18^ compared with housed individuals.^11^ Housing insecure individuals are often exposed to hazardous, unsanitary, and ever-changing living spaces, thus increasing their vulnerability to injury and infection. Furthermore, substance abuse and mental illness among those who are homeless predispose them to injury, assault, and infection or abscess due to IV drug use.^20,37^ Infections in homeless patients were attributed to a lack of hygiene, crowded living conditions, and concurrent skin pathologies.^29^ These patients are also more likely to forego medical care because of competing priorities to find food and shelter and not knowing where to go for care.^29^ Because of their lack of access to health care and exposure to hazardous environments, homeless children were more prone to musculoskeletal injury and disease.^30,35^ Overall, we found that our hypothesis was validated. Across these studies, homeless patients had a greater burden of musculoskeletal injury, mediated by circumstantial factors such as hazardous, ever-changing living spaces, substance abuse, and mental illness. In addition, many homeless patients are unable to receive adequate follow-up treatment because of lack of primary care physicians and overreliance on EDs that are not equipped for follow-up of musculoskeletal injuries.

Many homeless individuals are uninsured, whereas others cannot find child care.^3^ Because of their transient situation, homeless patients often do not have access to a regular primary care provider, and many often have negative care experiences due to social biases against homelessness.^39^ Even when homeless patients have access to primary care, they are often still likely to go to the ED because of familiarity, accessibility, or experiences with prejudice from primary care providers. As a result, homeless patients either lack knowledge of where to access care or are reluctant to access care. This predisposes them to using ED services for medical care in situations where the ED cannot properly meet their medical needs. In 2018, Zuccaro et al^38^ found that homeless patients presented to EDs for traumatic injuries and outpatient services, such as orthopaedics, because they lacked access to other healthcare options. The ED was often unable to meet the surgical needs of these patients in the same way that outpatient services could. Rafael Arceo et al^40^ also found that homeless individuals experience ballistic lower extremity fractures that were treated in the ED than nonhomeless individuals.

In addition, homeless patients have generally demonstrated poor follow-up after receiving medical care, especially within the field of orthopaedic surgery.^38^ Homeless patients have reported missing their follow-up appointments, especially after undergoing surgery, owing to barriers such as incarceration, lack of stable housing, or access to a clean environment and hygiene products.^22,25^ These issues may stem from homeless patients' overreliance on the ED and unstable living conditions that cause them to make follow-ups, especially for orthopaedic procedures, a lower priority. After undergoing surgery, homeless patients may also be unable to follow wound care guidance owing to their lack of stable housing or access to hygiene products.^20^ High prevalence of mental illness also effects follow-up care. Patients with mental illness can have difficulty understanding and following postoperative instructions, which may result in missed follow-up appointments.^20^ Clinicians should be aware of the obstacles homeless patients face in attending their follow-up appointments or adhering to postsurgery instructions. Clinicians should also be cautious with postoperative pain medication in this patient population and ensure that patients can safely and adequately take their prescribed medications. ED physicians should try to provide medications that patients can easily take in unstable living situations, such as medications that only have to be taken once per day.

Interventional studies on homeless populations can provide guidance for managing their care. For example, for outbreaks of invasive infections, mass antibiotic distribution efforts in homeless shelters can help reduce to the spread of disease.^29^ Physicians could also work with homeless shelters to ensure that patients have warm clothing, socks, and sturdy shoes to protect their feet from exposure to the elements, especially in colder months. In addition, community homeless clinics that offer massage therapy can reduce patient's pain and disability.^36^ All these interventions involve the direct delivery of care to homeless patients. In addition, in 2017, Bennet et al^15^ found that after total joint replacement in 37 homeless veterans, 73% of patients were able to be permanently housed, likely because of reduced pain, increased mobility, and access to resources through the Department of Veterans Affairs at the time of hospitalization. The study noted good follow-up (92%) despite homelessness generally being considered a contraindication to joint replacement surgery.

Our results are consistent with previous case reports, narratives, and studies suggesting a high burden of musculoskeletal conditions among homeless individuals. Despite the prevalence of these conditions, specific information regarding orthopaedic injury, treatment, and recovery within this population is unavailable: only one study specified which orthopaedic procedure was done to treat the musculoskeletal condition.^15^ Future studies should address musculoskeletal issues in underserved and homeless populations longitudinally and further explore factors such as preexisting conditions, race, housing status, and access to resources and follow-up care. Comparative studies between homeless and housed individuals could yield more information to target interventions for these populations.

There are several limitations to this study. First, only 29 articles were identified, most of which were published in the United States. Therefore, the findings may not be representative of musculoskeletal conditions among all homeless individuals, especially those in different countries. The low-reported quality of several studies, including lack of details on the specific musculoskeletal condition patients had, or self-reported musculoskeletal conditions from questionnaires, limit overall conclusions that can be drawn. There may also be non-published data in government health policy reports that could address issues such as poor follow up, mental health problems and overreliance on EDs. Second, there is room to improve quality of research reports on homeless individuals with musculoskeletal conditions. Many studies did not provide clear outcomes or study objectives, had limited information regarding study participants, and were insufficiently powered to detect any clinically significant conditions. Finally, many articles did not account for other social determinants of health, such as drug addiction, incarceration, lesbian, gay, bisexual, transgender and queer or questioning (LGBTQ) status, and chronic illnesses that could affect homeless patients and increase the risk of musculoskeletal injury. Further research is needed to better address the multifactorial aspects of homelessness and how they relate to orthopaedic injuries.

Orthopaedic musculoskeletal conditions are common among both adults and children experiencing homelessness. This study is a synthesis of what is currently known in the literature regarding musculoskeletal diseases and injuries among homeless persons. Homeless individuals often have inadequate access to care and thus must rely on the ED for traumatic injuries. These findings have important implications for surgeons and public health officials and highlight the need for evidence-based interventions and increased follow-up. Targeted efforts and better tracking of follow-up and ED usage could improve health outcome for homeless individuals and reduce the need for costly late-stage interventions by providing early and more consistent care.
